# Effectiveness of 2, 3, and 4 COVID-19 mRNA Vaccine Doses Among Immunocompetent Adults During Periods when SARS-CoV-2 Omicron BA.1 and BA.2/BA.2.12.1 Sublineages Predominated — VISION Network, 10 States, December 2021–June 2022

**DOI:** 10.15585/mmwr.mm7129e1

**Published:** 2022-07-22

**Authors:** Ruth Link-Gelles, Matthew E. Levy, Manjusha Gaglani, Stephanie A. Irving, Melissa Stockwell, Kristin Dascomb, Malini B. DeSilva, Sarah E. Reese, I-Chia Liao, Toan C. Ong, Shaun J. Grannis, Charlene McEvoy, Palak Patel, Nicola P. Klein, Emily Hartmann, Edward Stenehjem, Karthik Natarajan, Allison L. Naleway, Kempapura Murthy, Suchitra Rao, Brian E. Dixon, Anupam B. Kharbanda, Akintunde Akinseye, Monica Dickerson, Ned Lewis, Nancy Grisel, Jungmi Han, Michelle A. Barron, William F. Fadel, Margaret M. Dunne, Kristin Goddard, Julie Arndorfer, Deepika Konatham, Nimish R. Valvi, J. C. Currey, Bruce Fireman, Chandni Raiyani, Ousseny Zerbo, Chantel Sloan-Aagard, Sarah W. Ball, Mark G. Thompson, Mark W. Tenforde

**Affiliations:** ^1^CDC COVID-19 Emergency Response Team; ^2^Westat, Rockville, Maryland.; ^3^Baylor Scott & White Health, Temple, Texas; ^4^Texas A&M University College of Medicine, Temple, Texas; ^5^Center for Health Research, Kaiser Permanente Northwest, Portland, Oregon; ^6^Division of Child and Adolescent Health, Department of Pediatrics, Columbia University Vagelos College of Physicians and Surgeons, New York, New York; ^7^Department of Population and Family Health, Columbia University Mailman School of Public Health, New York, New York; ^8^New York Presbyterian Hospital, New York, New York; ^9^Division of Infectious Diseases and Clinical Epidemiology, Intermountain Healthcare, Salt Lake City, Utah; ^10^HealthPartners Institute, Minneapolis, Minnesota; ^11^School of Medicine, University of Colorado Anschutz Medical Campus, Aurora, Colorado; ^12^Center for Biomedical Informatics, Regenstrief Institute, Indianapolis, Indiana; ^13^School of Medicine, Indiana University, Indianapolis, Indiana; ^14^Kaiser Permanente Vaccine Study Center, Kaiser Permanente Northern California Division of Research, Oakland, California; ^15^Paso Del Norte Health Information Exchange, El Paso, Texas; ^16^Department of Biomedical Informatics, Columbia University Irving Medical Center, New York, New York; ^17^Fairbanks School of Public Health, Indiana University, Indianapolis, Indiana; ^18^Children's Minnesota, Minneapolis, Minnesota; ^19^Brigham Young University Department of Public Health, Provo, Utah.

The Omicron variant (B.1.1.529) of SARS-CoV-2, the virus that causes COVID-19, was first identified in the United States in November 2021, with the BA.1 sublineage (including BA.1.1) causing the largest surge in COVID-19 cases to date. Omicron sublineages BA.2 and BA.2.12.1 emerged later and by late April 2022, accounted for most cases.* Estimates of COVID-19 vaccine effectiveness (VE) can be reduced by newly emerging variants or sublineages that evade vaccine-induced immunity (*1*), protection from previous SARS-CoV-2 infection in unvaccinated persons (*2*), or increasing time since vaccination (*3*). Real-world data comparing VE during the periods when the BA.1 and BA.2/BA.2.12.1 predominated (BA.1 period and BA.2/BA.2.12.1 period, respectively) are limited. The VISION network^†^ examined 214,487 emergency department/urgent care (ED/UC) visits and 58,782 hospitalizations with a COVID-19–like illness^§^ diagnosis among 10 states during December 18, 2021–June 10, 2022, to evaluate VE of 2, 3, and 4 doses of mRNA COVID-19 vaccines (BNT162b2 [Pfizer-BioNTech] or mRNA-1273 [Moderna]) compared with no vaccination among adults without immunocompromising conditions. VE against COVID-19–associated hospitalization 7–119 days and ≥120 days after receipt of dose 3 was 92% (95% CI = 91%–93%) and 85% (95% CI = 81%–89%), respectively, during the BA.1 period, compared with 69% (95% CI = 58%–76%) and 52% (95% CI = 44%–59%), respectively, during the BA.2/BA.2.12.1 period. Patterns were similar for ED/UC encounters. Among adults aged ≥50 years, VE against COVID-19–associated hospitalization ≥120 days after receipt of dose 3 was 55% (95% CI = 46%–62%) and ≥7 days (median = 27 days) after a fourth dose was 80% (95% CI = 71%–85%) during BA.2/BA.2.12.1 predominance. Immunocompetent persons should receive recommended COVID-19 booster doses to prevent moderate to severe COVID-19, including a first booster dose for all eligible persons and second booster dose for adults aged ≥50 years at least 4 months after an initial booster dose. Booster doses should be obtained immediately when persons become eligible.^¶^

A 2-dose primary COVID-19 mRNA vaccination series followed by a third (booster) dose at least 5 months after dose 2 is recommended for adults aged ≥18 years without immunocompromising conditions. On March 29, 2022, an additional booster dose (dose 4) was authorized for immunocompetent adults aged ≥50 years at least 4 months after dose 3 (*4*). The VISION Network evaluated the effectiveness of 2, 3, or 4 mRNA vaccine doses during December 2021–June 2022, a period during which different sublineages of Omicron circulated in the United States. VISION methods have been described previously (*5*); briefly, eligible medical encounters include ED/UC visits and hospitalizations among adults with COVID-19–like illness and a SARS-CoV-2 molecular test during the 14 days before through 72 hours after the encounter. Variant predominance was defined as the period when a variant accounted for ≥75% of all sequenced specimens at a site (i.e., BA.1, December 2021–March 2022** and BA.2/BA.2.12.1, March–June 2022^††^). Dates when the prevalence of BA.1 declined to <75% of sequenced specimens and the prevalence of BA.2/BA.2.12.1 had not yet reached 75% were considered a “washout” period; encounters through June 10, 2022, were included unless BA.2/BA.2.12.1 prevalence declined to <75% at a site before that date. Patients were excluded if 1) a medical event occurred during the washout period; 2) a likely immunocompromising condition was present; 3) an mRNA vaccine dose was received before it was recommended^§§^; 4) any doses of a non–mRNA vaccine such as JNJ-78436735 (Janssen [Johnson & Johnson]) were received; 5) <14 days had elapsed since receipt of dose 2 or <7 days since receipt of dose 3 or dose 4; or 6) a previous SARS-CoV-2 infection was documented in the patient’s medical record before the index encounter (to reduce the influence of protection from previous infection).^¶¶^ VE was estimated using a test-negative case-control design, comparing the odds of being vaccinated (receipt of 2 doses ≥14 days before the encounter, 3 doses ≥7 days before the encounter, or 4 doses ≥7 days before the encounter) versus being unvaccinated (zero doses received) between persons with positive and negative SARS-CoV-2 test results, using multivariable logistic regression, weighted for inverse propensity to be vaccinated, and adjusted for age, calendar time of index date (days since January 1, 2021),*** study site, and local virus circulation. VE for 4 vaccine doses was assessed only for adults aged ≥50 years during the BA.2/BA.2.12.1 period, aligning with the March 29, 2022, authorization for the fourth dose. Nonoverlapping 95% CIs were considered statistically significant. Analyses were conducted using R software (version 4.1.2; R Foundation). The study was reviewed and approved by institutional review boards at participating sites or under reliance agreement with the institutional review board of Westat, Inc. This activity was conducted consistent with applicable federal law and CDC policy.^†††^

Among 214,487 ED/UC encounters with a COVID-19–like illness diagnosis that met inclusion criteria, 124,033 (57.8%) occurred during the BA.1 period, during which 40,801 (32.9%) patients had a positive SARS-CoV-2 test result; 90,454 (42.2%) occurred during the BA.2/BA.2.12.1 period, during which 10,177 (11.3%) had a positive SARS-CoV-2 test result. During the BA.1 period, 51,359 (41.4%) ED/UC patients were unvaccinated, 40,026 (32.3%) had received 2 mRNA vaccine doses, and 32,648 (26.3%) had received 3 doses (Table 1). During the BA.2/BA.2.12.1 period, 27,907 (30.9%) ED/UC patients were unvaccinated; 22,657 (25.0%) had received 2 mRNA vaccine doses, 35,796 (39.6%) had received 3 doses; and 4,094 (4.5%) had received 4 doses. Receipt of 3 versus 2 doses was associated with a higher VE against an ED/UC encounter during both periods, and VE was higher during the BA.1 period than the BA.2/BA.2.12.1 period (Table 2). During the BA.1 period, VE declined to 73% ≥120 days (median = 132 days) after the third vaccine dose; during the BA.2/BA.12.1 period, VE declined to 26% at ≥120 days (median = 166 days) after the third dose.

**TABLE 1:** Characteristics of emergency department and urgent care encounters among adults aged ≥18 years with COVID-19–like illness,* by Omicron subvariant–predominant period,^†^^,^^§^ mRNA COVID-19 vaccination status, and SARS-CoV-2 test result — 10 states, December 2021–June 2022

Characteristic	Total no. (column %)	mRNA COVID-19 vaccination status^¶^							Positive test result*	
		No. (row %)						SMD**	No. (row %)	SMD**
		Unvaccinated	2 doses		3 doses		4 doses			
			14–149 days earlier	≥150 days earlier	7–119 days earlier	≥120 days earlier	≥7 days earlier			
**Omicron BA.1**–**predominant period^†^**										
**All ED or UC events**	**124,033 (100.0)**	51,359 (41.4)	7,286 (5.9)	32,740 (26.4)	29,333 (23.6)	3,315 (2.7)	N/A	—	40,801 (32.9)	—
**Site**										
Baylor Scott & White Health	**29,978 (24.2)**	17,365 (57.9)	1,544 (5.2)	7,799 (26.0)	2,970 (9.9)	300 (1.0)	—	0.745	13,279 (44.3)	0.342
Columbia University	**3,116 (2.5)**	1,600 (51.3)	333 (10.7)	740 (23.7)	432 (13.9)	11 (0.4)	—		956 (30.7)	
HealthPartners	**12,579 (10.1)**	3,435 (27.3)	730 (5.8)	3,247 (25.8)	4,720 (37.5)	447 (3.6)	—		3,820 (30.4)	
Intermountain Healthcare	**26,950 (21.7)**	9,717 (36.1)	2,020 (7.5)	7,398 (27.5)	6,844 (25.4)	971 (3.6)	—		6,696 (24.8)	
KPNC	**20,383 (16.4)**	3,862 (18.9)	1,274 (6.3)	5,952 (29.2)	8,411 (41.3)	884 (4.3)	—		5,252 (25.8)	
KPNW	**7,929 (6.4)**	2,417 (30.5)	385 (4.9)	2,166 (27.3)	2,544 (32.1)	417 (5.3)	—		2,686 (33.9)	
PHIX	**1,243 (1.0)**	647 (52.1)	54 (4.3)	322 (25.9)	196 (15.8)	24 (1.9)	—		318 (25.6)	
Regenstrief Institute	**14,003 (11.3)**	8,007 (57.2)	682 (4.9)	2,968 (21.2)	2,213 (15.8)	133 (0.9)	—		4,986 (35.6)	
University of Colorado	**7,852 (6.3)**	4,309 (54.9)	264 (3.4)	2,148 (27.4)	1,003 (12.8)	128 (1.6)	—		2,808 (35.8)	
**Age group, yrs**										
18–49	**63,406 (51.1)**	33,003 (52.1)	4,909 (7.7)	16,313 (25.7)	8,755 (13.8)	426 (0.7)	—	0.678	23,073 (36.4)	0.219
50–65	**24,832 (20.0)**	9,229 (37.2)	1,415 (5.7)	7,458 (30.0)	6,305 (25.4)	425 (1.7)	—		8,460 (34.1)	
65–74	**15,978 (12.9)**	4,646 (29.1)	507 (3.2)	3,901 (24.4)	5,953 (37.3)	971 (6.1)	—		4,459 (27.9)	
75–84	**12,584 (10.1)**	2,940 (23.4)	302 (2.4)	3,205 (25.5)	5,179 (41.2)	958 (7.6)	—		3,224 (25.6)	
≥85	**7,233 (5.8)**	1,541 (21.3)	153 (2.1)	1,863 (25.8)	3,141 (43.4)	535 (7.4)	—		1,585 (21.9)	
**Sex**										
Male	**50,479 (40.7)**	22,531 (44.6)	2,536 (5.0)	12,433 (24.6)	11,574 (22.9)	1,405 (2.8)	—	0.107	17,286 (34.2)	0.051
Female	**73,554 (59.3)**	28,828 (39.2)	4,750 (6.5)	20,307 (27.6)	17,759 (24.1)	1,910 (2.6)	—		23,515 (32.0)	
**Race or ethnicity**										
White, NH	**74,613 (60.2)**	28,365 (38.0)	3,746 (5.0)	19,754 (26.5)	20,145 (27.0)	2,603 (3.5)	—	0.356	21,430 (28.7)	0.255
Black, NH	**15,395 (12.4)**	8,547 (55.5)	1,295 (8.4)	3,505 (22.8)	1,902 (12.4)	146 (0.9)	—		6,529 (42.4)	
Hispanic	**19,508 (15.7)**	8,893 (45.6)	1,451 (7.4)	5,489 (28.1)	3,446 (17.7)	229 (1.2)	—		7,481 (38.3)	
Other,**^††^** NH	**9,368 (7.6)**	2,802 (29.9)	522 (5.6)	2,754 (29.4)	3,011 (32.1)	279 (3.0)	—		3,061 (32.7)	
Unknown	**5,149 (4.2)**	2,752 (53.4)	272 (5.3)	1,238 (24.0)	829 (16.1)	58 (1.1)	—		2,300 (44.7)	
**Chronic respiratory condition at discharge^§§^**										
No	**103,754 (83.7)**	43,204 (41.6)	6,287 (6.1)	27,363 (26.4)	24,303 (23.4)	2,597 (2.5)	—	0.065	34,674 (33.4)	0.054
Yes	**20,279 (16.3)**	8,155 (40.2)	999 (4.9)	5,377 (26.5)	5,030 (24.8)	718 (3.5)	—		6,127 (30.2)	
**Chronic nonrespiratory condition at discharge^¶¶^**										
No	**91,182 (73.5)**	38,741 (42.5)	5,749 (6.3)	24,157 (26.5)	20,551 (22.5)	1,984 (2.2)	—	0.145	31,826 (34.9)	0.154
Yes	**32,851 (26.5)**	12,618 (38.4)	1,537 (4.7)	8,583 (26.1)	8,782 (26.7)	1,331 (4.1)	—		8,975 (27.3)	
**Omicron BA.2/BA.2.12.1–predominant period^§^**										
**All ED or UC events**	**90,454 (100.0)**	27,907 (30.9)	1,774 (2.0)	20,883 (23.1)	9,142 (10.1)	26,654 (29.5)	4,094 (4.5)	—	10,177 (11.3)	—
**Site**										
Baylor Scott & White Health	**12,976 (14.3)**	6,786 (52.3)	188 (1.4)	3,687 (28.4)	501 (3.9)	1,720 (13.3)	94 (0.7)	0.925	1,155 (8.9)	0.296
Columbia University	**3,430 (3.8)**	1,452 (42.3)	130 (3.8)	937 (27.3)	344 (10.0)	551 (16.1)	16 (0.5)		232 (6.8)	
HealthPartners	**15,234 (16.8)**	3,269 (21.5)	346 (2.3)	2,868 (18.8)	1,821 (12.0)	5,944 (39.0)	986 (6.5)		2,057 (13.5)	
Intermountain Healthcare	**17,134 (18.9)**	5,262 (30.7)	469 (2.7)	4,359 (25.4)	1,654 (9.7)	4,986 (29.1)	404 (2.4)		2,318 (13.5)	
KPNC	**20,732 (22.9)**	2,531 (12.2)	374 (1.8)	4,114 (19.8)	3,278 (15.8)	8,446 (40.7)	1,989 (9.6)		1,670 (8.1)	
KPNW	**7,211 (8.0)**	1,588 (22.0)	110 (1.5)	1,464 (20.3)	894 (12.4)	2,695 (37.4)	460 (6.4)		1,084 (15.0)	
PHIX	**709 (0.8)**	338 (47.7)	13 (1.8)	176 (24.8)	59 (8.3)	113 (15.9)	10 (1.4)		43 (6.1)	
Regenstrief Institute	**6,064 (6.7)**	3,188 (52.6)	95 (1.6)	1,299 (21.4)	341 (5.6)	1,103 (18.2)	38 (0.6)		575 (9.5)	
University of Colorado	**6,964 (7.7)**	3,493 (50.2)	49 (0.7)	1,979 (28.4)	250 (3.6)	1,096 (15.7)	97 (1.4)		1,043 (15.0)	
**Age group, yrs**										
18–49	**42,569 (47.1)**	18,429 (43.3)	1,192 (2.8)	11,203 (26.3)	4,132 (9.7)	7,613 (17.9)	0 (0.0)	0.778	5,074 (11.9)	0.099
50–65	**17,598 (19.5)**	4,755 (27.0)	317 (1.8)	4,253 (24.2)	2,232 (12.7)	5,355 (30.4)	686 (3.9)		2,087 (11.9)	
65–74	**12,909 (14.3)**	2,271 (17.6)	137 (1.1)	2,437 (18.9)	1,185 (9.2)	5,542 (42.9)	1337 (10.4)		1,253 (9.7)	
75–84	**11,032 (12.2)**	1,591 (14.4)	71 (0.6)	1,902 (17.2)	994 (9.0)	5,130 (46.5)	1344 (12.2)		1,174 (10.6)	
≥85	**6,346 (7.0)**	861 (13.6)	57 (0.9)	1,088 (17.1)	599 (9.4)	3,014 (47.5)	727 (11.5)		589 (9.3)	
**Sex**										
Male	**36,191 (40.0)**	11,836 (32.7)	631 (1.7)	8,014 (22.1)	3,406 (9.4)	10,449 (28.9)	1,855 (5.1)	0.090	4,091 (11.3)	0.004
Female	**54,263 (60.0)**	16,071 (29.6)	1,143 (2.1)	12,869 (23.7)	5,736 (10.6)	16,205 (29.9)	2,239 (4.1)		6,086 (11.2)	
**Race or ethnicity**										
White, NH	**55,447 (61.3)**	15,386 (27.7)	799 (1.4)	12,474 (22.5)	5,296 (9.6)	18,410 (33.2)	3,082 (5.6)	0.361	6,471 (11.7)	0.128
Black, NH	**9,797 (10.8)**	4,405 (45.0)	368 (3.8)	2,272 (23.2)	898 (9.2)	1,644 (16.8)	210 (2.1)		1,033 (10.5)	
Hispanic	**13,939 (15.4)**	4,780 (34.3)	396 (2.8)	3,693 (26.5)	1,642 (11.8)	3,076 (22.1)	352 (2.5)		1,217 (8.7)	
Other,**^††^** NH	**8,040 (8.9)**	1,769 (22.0)	160 (2.0)	1,670 (20.8)	1,096 (13.6)	2,927 (36.4)	418 (5.2)		1,003 (12.5)	
Unknown	**3,231 (3.6)**	1,567 (48.5)	51 (1.6)	774 (24.0)	210 (6.5)	597 (18.5)	32 (1.0)		453 (14.0)	
**Chronic respiratory condition at discharge^§§^**										
No	**75,947 (84.0)**	23,604 (31.1)	1,474 (1.9)	17,438 (23.0)	7,708 (10.1)	22,242 (29.3)	3,481 (4.6)	0.024	9,149 (12.0)	0.197
Yes	**14,507 (16.0)**	4,303 (29.7)	300 (2.1)	3,445 (23.7)	1,434 (9.9)	4,412 (30.4)	613 (4.2)		1,028 (7.1)	
**Chronic nonrespiratory condition at discharge^¶¶^**										
No	**67,691 (74.8)**	21,424 (31.6)	1,359 (2.0)	15,621 (23.1)	6,903 (10.2)	19,378 (28.6)	3,006 (4.4)	0.050	8,549 (12.6)	0.255
Yes	**22,763 (25.2)**	6,483 (28.5)	415 (1.8)	5,262 (23.1)	2,239 (9.8)	7,276 (32.0)	1,088 (4.8)		1,628 (7.2)	

**Abbreviations:** ED = emergency department; ICD-9 = *International Classification of diseases, Ninth Revision*; ICD-10 = *International Classification of diseases, Tenth Revision*; KPNC = Kaiser Permanente Northern California; KPNW = Kaiser Permanente Northwest; N/A = not applicable; NH = non-Hispanic; PHIX = Paso del Norte Health Information Exchange; RT-PCR = reverse transcription–polymerase reaction; SMD = standardized mean or proportion difference; UC = urgent care.

* Medical events with a discharge code consistent with COVID-19–like illness were included; using ICD-9 and ICD-10 codes, COVID-19–like illness diagnoses included acute respiratory illness (e.g., respiratory failure or pneumonia) or related signs or symptoms (e.g., cough, fever, dyspnea, vomiting, or diarrhea). Clinician-ordered molecular assays (e.g., real-time RT-PCR) for SARS-CoV-2 occurring ≤14 days before to <72 hours after the encounter date were included.

**^†^** Partners contributing data on medical events during dates of estimated ≥75% Omicron BA.1 predominance were in California (Dec 21, 2021–Mar 6, 2022), Colorado (Dec 25, 2021–Mar 12, 2022), Indiana (Dec 31, 2021–Mar 4, 2022), Minnesota and Wisconsin (Jan 1–Mar 5, 2022), New York (Dec 18, 2021–Feb 26, 2022), Oregon and Washington (Jan 1–Mar 12, 2022), Texas (Baylor Scott & White Health [Dec 18, 2021–Mar 5, 2022] and PHIX [Jan 8–Mar 19, 2022]), and Utah (Dec 27, 2021–Mar 19, 2022).

**^§^** Partners contributing data on medical events during dates of estimated ≥75% Omicron BA.2/BA.2.12.1 predominance were in California (Mar 25–Jun 10, 2022), Colorado (Apr 9–Jun 4, 2022), Indiana (Mar 19–Jun 10, 2022), Minnesota and Wisconsin (Apr 9–Jun 4, 2022), New York (Mar 26–Jun 10, 2022), Oregon and Washington (Apr 9–Jun 10, 2022), Texas (Baylor Scott & White Health [Mar 26–Jun 4, 2022] and PHIX [Apr 23–Jun 10, 2022]), and Utah (Mar 28–Jun 10, 2022).

^¶^ Vaccination was defined as having received the listed number of doses of an mRNA-based COVID-19 vaccine within the specified range of number of days before the medical event index date, which was the date of respiratory specimen collection associated with the most recent positive or negative SARS-CoV-2 test result before the medical event or the admission date if testing only occurred after the admission.

** An absolute SMD ≥0.20 indicates a nonnegligible difference in variable distributions between medical events for vaccinated versus unvaccinated patients or for patients with SARS-CoV-2–positive test result versus those with SARS-CoV-2–negative results. For mRNA COVID-19 vaccination status, a single SMD was calculated by averaging the absolute SMDs obtained from pairwise comparisons of each vaccinated category versus unvaccinated; more specifically as the average of the absolute value of the SMDs for 1) vaccinated with 2 doses 14–149 days earlier versus unvaccinated, 2) vaccinated with 2 doses ≥150 days earlier versus unvaccinated, 3) vaccinated with 3 doses 7–119 days earlier versus unvaccinated, 4) vaccinated with 3 doses ≥120 days earlier versus unvaccinated, and 5) vaccinated with 4 doses ≥7 days earlier versus unvaccinated.

**^††^** Other race includes Asian, Native Hawaiian or other Pacific islander, American Indian or Alaska Native, Other, and multiple races.

**^§§^** Chronic respiratory condition was defined as the presence of discharge code for asthma, chronic obstructive pulmonary disease, or other lung disease using ICD-9 or ICD-10 diagnosis codes.

^¶¶^ Chronic nonrespiratory condition was defined as the presence of discharge code for heart failure, ischemic heart disease, hypertension, other heart disease, stroke, other cerebrovascular disease, diabetes type I or II, other diabetes, metabolic disease, clinical obesity, clinically underweight, renal disease, liver disease, blood disorder, immunosuppression, organ transplant, cancer, dementia, neurologic disorder, musculoskeletal disorder, or Down syndrome using ICD-9 and ICD-10 diagnosis codes.

**TABLE 2:** mRNA COVID-19 vaccine effectiveness* against laboratory-confirmed COVID-19–associated^†^ emergency department and urgent care encounters and hospitalizations among adults aged ≥18 years, by Omicron–predominant period, age group, number and timing of vaccine doses,^§^ and median interval since last dose — VISION Network, 10 states, December 2021–June 2022

Encounter type	Omicron BA.1–predominant period^¶^				Omicron BA.2/BA.2.12.1–predominant period**			
	Total	No. (%) of positive test results^†^	Median interval since last dose, days (IQR)	VE %* (95% CI)	Total	No. (%) of positive test results^†^	Median interval since last dose days (IQR)	VE %* (95% CI)
**ED or UC, age group (days since last dose)**								
**All ages, yrs**								
Unvaccinated (Ref)	**51,359**	23,175 (45.1)	—	—	**27,907**	3,501 (12.6)	—	—
2 doses (14–149)	**7,286**	2,377 (32.6)	107 (76–129)	47 (44–50)	**1,774**	110 (6.2)	104 (71–128)	51 (38–60)
2 doses (≥150)	**32,740**	11,365 (34.7)	267 (232–306)	39 (37–41)	**20,883**	2,584 (12.4)	352 (278–398)	12 (7–17)
3 doses (7–119)	**29,333**	3,667 (12.5)	66 (41–89)	84 (83–85)	**9,142**	441 (4.8)	94 (72–108)	56 (51–61)
3 doses (≥120)	**3,315**	217 (6.5)	132 (125–142)	73 (68–77)	**26,654**	3,186 (11.9)	166 (145–190)	26 (21–30)
**18–49 yrs**								
Unvaccinated (Ref)	**33,003**	14,236 (43.1)	—	—	**18,429**	2,269 (12.3)	—	—
2 doses (14–149)	**4,909**	1,621 (33.0)	106 (76–129)	40 (36–44)	**1,192**	75 (6.3)	105 (72–129)	47 (31–60)
2 doses (≥150)	**16,313**	5,918 (36.3)	252 (220–288)	24 (21–28)	**11,203**	1,427 (12.7)	332 (254–379)	7 (0–14)
3 doses (7–119)	**8,755**	1,259 (14.4)	55 (33–79)	76 (75–78)	**4,132**	207 (5.0)	91 (69–107)	55 (47–62)
3 doses (≥120)	**426**	39 (9.2)	130 (124–141)	29 (−1–50)	**7,613**	1,096 (14.4)	159 (140–182)	17 (10–25)
**≥50 yrs**								
Unvaccinated (Ref)	**18,356**	8,939 (48.7)	—	—	**9,478**	1,232 (13.0)	—	—
2 doses (14–149)	**2,377**	756 (31.8)	109 (77–129)	59 (54–63)	**582**	35 (6.0)	102 (68–128)	59 (40–71)
2 doses (≥150)	**16,427**	5,447 (33.2)	283 (248–316)	52 (50–54)	**9,680**	1,157 (11.9)	376 (319–414)	18 (10–26)
3 doses (7–119)	**20,578**	2,408 (11.7)	71 (46–93)	87 (86–88)	**5,010**	234 (4.7)	96 (73–109)	58 (51–64)
3 doses (≥120)	**2,889**	178 (6.2)	133 (125–143)	81 (77–84)	**19,041**	2,090 (11.0)	170 (147–193)	32 (26–38)
4 doses (≥7)^††^	**N/A**	—	—	—	**4,094**	355 (8.7)	28 (17–42)	66 (60–71)
**Hospitalization, age group (days since last dose)**								
**All ages, yrs**								
Unvaccinated (Ref)	**14,742**	6,829 (46.3)	—	—	**6,682**	494 (7.4)	—	—
2 doses (14–149)	**1,236**	297 (24.0)	105 (73–129)	68 (63–73)	**343**	12 (3.5)	102 (71–128)	57 (19–77)
2 doses (≥150)	**8,850**	2,542 (28.7)	289 (252–322)	61 (58–63)	**5,118**	393 (7.7)	371 (308–413)	24 (12–35)
3 doses (7–119)	**9,146**	786 (8.6)	72 (47–93)	92 (91–93)	**2,350**	72 (3.1)	94 (74–108)	69 (58–76)
3 doses (≥120)	**1,425**	80 (5.6)	132 (125–142)	85 (81–89)	**7,686**	519 (6.8)	168 (146–191)	52 (44–59)
**18–49 yrs^§§^**								
Unvaccinated (Ref)	**4,057**	1,515 (37.3)	—	—	**—**	—	—	—
2 doses (14–149)	**392**	83 (21.2)	101 (67–127)	64 (52–73)	**—**	—	—	—
2 doses (≥150)	**1,304**	329 (25.2)	258 (226–294)	52 (43–59)	**—**	—	—	—
3 doses (7–119)	**812**	53 (6.5)	57 (36–81)	91 (87–94)	**—**	—	—	—
3 doses (≥120)	**56**	1 (1.8)	133 (126–142)	94 (62–99)	**—**	—	—	—
**≥50 yrs^§§^**								
Unvaccinated (Ref)	**10,685**	5,314 (49.7)	—	—	**4,595**	393 (8.6)	—	—
2 doses (14–149)	**844**	214 (25.4)	108 (76–129)	71 (65–75)	**—**	—	—	—
2 doses (≥150)	**7,546**	2,213 (29.3)	294 (259–325)	63 (60–66)	**4,139**	352 (8.5)	381 (325–418)	22 (8–34)
3 doses (7–119)	**8,334**	733 (8.8)	73 (49–94)	92 (91–93)	**1,957**	57 (2.9)	95 (74–108)	73 (63–81)
3 doses (≥120)	**1,369**	79 (5.8)	132 (125–142)	86 (82–89)	**7,113**	480 (6.8)	169 (147–191)	55 (46–62)
4 doses (≥7)^††^	**N/A**	—	—	—	**1,204**	74 (6.2)	27 (17–41)	80 (71–85)

**Abbreviations**: ED = emergency department; ICD-9 = *International Classification of Diseases, Ninth Revision*; ICD-10 = *International Classification of Diseases, Tenth Revision*; N/A = not applicable; PHIX = Paso Del Norte Health Information Exchange; Ref = referent group; RT-PCR = reverse transcription–polymerase chain reaction; UC = urgent care; VE = vaccine effectiveness.

* VE was calculated as ([1−odds ratio] x 100%), estimated using a test-negative design, adjusted for age, geographic region, calendar time (days since January 1, 2021), and local virus circulation (percentage of SARS-CoV-2–positive results from testing within the counties surrounding the facility on the date of the encounter) and weighted for inverse propensity to be vaccinated or unvaccinated (calculated separately for each set of VE estimates among ED or UC encounters and hospitalizations by Omicron–predominant period and age group). Generalized boosted regression trees were used to estimate the propensity to be vaccinated based on sociodemographic characteristics, underlying medical conditions, and facility characteristics.

^†^ Medical events with a discharge code consistent with COVID-19–like illness were included. COVID-19–like illness diagnoses included acute respiratory illness (e.g., respiratory failure or pneumonia) or related signs or symptoms (e.g., cough, fever, dyspnea, vomiting, or diarrhea) using ICD-9 and ICD-10 codes. Clinician-ordered molecular assays (e.g., real-time RT-PCR) for SARS-CoV-2 occurring ≤14 days before to <72 hours after the encounter date were included.

^§^ Vaccination was defined as having received the listed number of doses of an mRNA-based COVID-19 vaccine within the specified range of number of days before the medical event index date, which was the date of respiratory specimen collection associated with the most recent positive or negative SARS-CoV-2 test result before the medical event or the admission date if testing only occurred after the admission.

^¶^ Partners contributing data on medical events during dates of estimated ≥75% Omicron BA.1 predominance were in California (Dec 21, 2021–Mar 6, 2022), Colorado (Dec 25, 2021–Mar 12, 2022), Indiana (Dec 31, 2021–Mar 4, 2022), Minnesota and Wisconsin (Jan 1–Mar 5, 2022), New York (Dec 18, 2021–Feb 26, 2022), Oregon and Washington (Jan 1–Mar 12, 2022), Texas (Baylor Scott & White Health [Dec 18, 2021–Mar 5, 2022] and PHIX [Jan 8–Mar 19, 2022]), and Utah (Dec 27, 2021–Mar 19, 2022).

** Partners contributing data on medical events during dates of estimated ≥75% Omicron BA.2/BA.2.12.1 predominance were in California (Mar 25–Jun 10, 2022), Colorado (Apr 9–Jun 4, 2022), Indiana (Mar 19–Jun 10, 2022), Minnesota and Wisconsin (Apr 9–Jun 4, 2022), New York (Mar 26–Jun 10, 2022), Oregon and Washington (Apr 9–Jun 10, 2022), Texas (Baylor Scott & White Health [Mar 6–Jun 4, 2022 and PHIX [Apr 23–Jun 10, 2022]), and Utah (Mar 28–Jun 10, 2022).

^††^ For estimation of 4-dose mRNA VE among patients aged ≥50 years during the Omicron BA.2/BA.2.12.1–predominant period, unvaccinated patients whose medical event index date was before Apr 5, 2022 were excluded from the referent group (1,836 ED or UC encounters and 999 hospitalizations excluded among unvaccinated patients) because the earliest medical event index date included among 4-dose mRNA-vaccinated patients was 7 days after Mar 29, 2022 when a second booster mRNA vaccine dose (fourth dose) was first included in recommendations for adults aged ≥50 years (at least 4 months after receiving a third mRNA dose).

^§§^ VE estimates with 95% CIs >50 percentage points are not shown because of imprecision.

Among 58,782 hospitalizations with a COVID-19–like illness diagnosis that met inclusion criteria, 35,399 (60.2%) occurred during the BA.1 period, during which 10,534 (29.8%) patients had a positive SARS-CoV-2 test result; 23,383 (17.9%) occurred during the BA.2/BA.2.12.1 period, during which 1,564 (6.7%) patients had a positive test result (Table 3). During the BA.1 period, 14,742 (41.6%) patients hospitalized with COVID-19–like illness were unvaccinated, 10,086 (28.5%) had received 2 mRNA vaccine doses, and 10,571 (29.9%) had received 3 doses. During the BA.2/BA.2.12.1 period, 6,682 (28.6%) patients hospitalized with COVID-19–like illness were unvaccinated, and 5,461 (23.4%), 10,036 (42.9%), and 1,204 (5.1%) had received 2, 3, and 4 mRNA vaccine doses, respectively. VE against COVID-19–associated hospitalization was 61% ≥150 days after dose 2 during the BA.1 period (median = 289 days) compared with 24% during the BA.2/BA.2.12.1 period (median = 371 days) (Table 2). VE associated with a third mRNA vaccine dose was higher than that associated with a second vaccine dose but declined during both periods at ≥120 days to 85% during the BA.1 period (median = 132 days) and 52% during the BA.2/BA.2.12.1 period (median = 168 days).

**TABLE 3:** Characteristics of hospitalizations among adults aged ≥18 years with COVID-19–like illness,* by Omicron subvariant–predominant period, mRNA COVID-19 vaccination status, and SARS-CoV-2 test result — 10 states, December 2021–June 2022

Characteristic	Total no. (column %)	mRNA COVID-19 vaccination status, no. of doses received^¶^							Positive test result*	
		No. (row %)						SMD**	No. (row %)	SMD**
		Unvaccinated	Days since last dose				4 doses			
			2 doses		3 doses					
			14–149	≥150	7–119	≥120	≥7			
**Omicron BA.1**–**predominant period^†^**										
**All hospitalizations**	**35,399 (100.0)**	14,742 (41.6)	1,236 (3.5)	8,850 (25.0)	9,146 (25.8)	1,425 (4.0)	N/A	—	10,534 (29.8)	—
**Site**										
Baylor Scott& White Health	**8,697 (24.6)**	4,480 (51.5)	324 (3.7)	2,528 (29.1)	1,190 (13.7)	175 (2.0)	—	0.551	2,904 (33.4)	0.218
Columbia University	**1,419 (4.0)**	668 (47.1)	94 (6.6)	367 (25.9)	274 (19.3)	16 (1.1)	—		536 (37.8)	
HealthPartners	**1,334 (3.8)**	378 (28.3)	40 (3.0)	262 (19.6)	586 (43.9)	68 (5.1)	—		322 (24.1)	
Intermountain Healthcare	**3,224 (9.1)**	1,159 (35.9)	148 (4.6)	701 (21.7)	985 (30.6)	231 (7.2)	—		756 (23.4)	
KPNC	**6,911 (19.5)**	1,501 (21.7)	219 (3.2)	1,748 (25.3)	3,036 (43.9)	407 (5.9)	—		1,940 (28.1)	
KPNW	**1,480 (4.2)**	539 (36.4)	56 (3.8)	288 (19.5)	478 (32.3)	119 (8.0)	—		360 (24.3)	
PHIX	**96 (0.3)**	64 (66.7)	1 (1.0)	19 (19.8)	11 (11.5)	1 (1.0)	—		45 (46.9)	
Regenstrief Institute	**8,980 (25.4)**	4,398 (49.0)	276 (3.1)	1,969 (21.9)	2,076 (23.1)	261 (2.9)	—		2,937 (32.7)	
University of Colorado	**3,258 (9.2)**	1,555 (47.7)	78 (2.4)	968 (29.7)	510 (15.7)	147 (4.5)	—		734 (22.5)	
**Age group, yrs**										
18–49	**6,621 (18.7)**	4,057 (61.3)	392 (5.9)	1,304 (19.7)	812 (12.3)	56 (0.8)	—	0.540	1,981 (29.9)	0.126
50–65	**7,783 (22.0)**	3,847 (49.4)	328 (4.2)	2,008 (25.8)	1,470 (18.9)	130 (1.7)	—		2,664 (34.2)	
65–74	**8,073 (22.8)**	3,059 (37.9)	233 (2.9)	2,041 (25.3)	2,325 (28.8)	415 (5.1)	—		2,370 (29.4)	
75–84	**7,654 (21.6)**	2,329 (30.4)	178 (2.3)	2,054 (26.8)	2,609 (34.1)	484 (6.3)	—		2,137 (27.9)	
≥85	**5,268 (14.9)**	1,450 (27.5)	105 (2.0)	1,443 (27.4)	1,930 (36.6)	340 (6.5)	—		1,382 (26.2)	
**Sex**										
Male	**17,164 (48.5)**	7,549 (44.0)	529 (3.1)	4,075 (23.7)	4,308 (25.1)	703 (4.1)	—	0.098	5,428 (31.6)	0.087
Female	**18,235 (51.5)**	7,193 (39.4)	707 (3.9)	4,775 (26.2)	4,838 (26.5)	722 (4.0)	—		5,106 (28.0)	
**Race or ethnicity**										
White, NH	**22,967 (64.9)**	8,837 (38.5)	697 (3.0)	5,843 (25.4)	6,479 (28.2)	1,111 (4.8)	—	0.285	6,224 (27.1)	0.199
Black, NH	**4,214 (11.9)**	2,279 (54.1)	212 (5.0)	976 (23.2)	676 (16.0)	71 (1.7)	—		1,474 (35.0)	
Hispanic	**3,781 (10.7)**	1,801 (47.6)	188 (5.0)	960 (25.4)	759 (20.1)	73 (1.9)	—		1,491 (39.4)	
Other,**^††^** NH	**2,601 (7.3)**	893 (34.3)	81 (3.1)	628 (24.1)	880 (33.8)	119 (4.6)	—		760 (29.2)	
Unknown	**1,836 (5.2)**	932 (50.8)	58 (3.2)	443 (24.1)	352 (19.2)	51 (2.8)	—		585 (31.9)	
**Chronic respiratory condition at discharge^§§^**										
No	**14,763 (41.7)**	6,116 (41.4)	555 (3.8)	3,693 (25.0)	3,818 (25.9)	581 (3.9)	—	0.023	3,482 (23.6)	0.254
Yes	**20,636 (58.3)**	8,626 (41.8)	681 (3.3)	5,157 (25.0)	5,328 (25.8)	844 (4.1)	—		7,052 (34.2)	
**Chronic nonrespiratory condition at discharge^¶¶^**										
No	**4,685 (13.2)**	2,516 (53.7)	166 (3.5)	958 (20.4)	949 (20.3)	96 (2.0)	—	0.200	1,522 (32.5)	0.050
Yes	**30,714 (86.8)**	12,226 (39.8)	1,070 (3.5)	7,892 (25.7)	8,197 (26.7)	1,329 (4.3)	—		9,012 (29.3)	
**Omicron BA.2/BA.2.12.1–predominant period^§^**										
**All hospitalizations**	**23,383 (100.0)**	6,682 (28.6)	343 (1.5)	5,118 (21.9)	2,350 (10.1)	7,686 (32.9)	1,204 (5.1)	—	1,564 (6.7)	—
**Site**										
Baylor Scott & White Health	**4,686 (20.0)**	2,128 (45.4)	55 (1.2)	1,417 (30.2)	227 (4.8)	813 (17.3)	46 (1.0)	0.945	196 (4.2)	0.268
Columbia University	**1,413 (6.0)**	491 (34.7)	48 (3.4)	316 (22.4)	169 (12.0)	375 (26.5)	14 (1.0)		81 (5.7)	
HealthPartners	**1,758 (7.5)**	329 (18.7)	37 (2.1)	261 (14.8)	204 (11.6)	760 (43.2)	167 (9.5)		120 (6.8)	
Intermountain Healthcare	**2,023 (8.7)**	571 (28.2)	35 (1.7)	446 (22.0)	179 (8.8)	733 (36.2)	59 (2.9)		167 (8.3)	
KPNC	**6,866 (29.4)**	677 (9.9)	87 (1.3)	1,164 (17.0)	1,095 (15.9)	3,105 (45.2)	738 (10.7)		584 (8.5)	
KPNW	**1,326 (5.7)**	356 (26.8)	17 (1.3)	210 (15.8)	165 (12.4)	488 (36.8)	90 (6.8)		86 (6.5)	
PHIX	**12 (0.1)**	7 (58.3)	0 (0.0)	3 (25.0)	0 (0.0)	2 (16.7)	0 (0.0)		1 (8.3)	
Regenstrief Institute	**3,947 (16.9)**	1,600 (40.5)	48 (1.2)	869 (22.0)	246 (6.2)	1,128 (28.6)	56 (1.4)		235 (6.0)	
University of Colorado	**1,352 (5.8)**	523 (38.7)	16 (1.2)	432 (32.0)	65 (4.8)	282 (20.9)	34 (2.5)		94 (7.0)	
**Age group, yrs**										
18–49	**4,162 (17.8)**	2,087 (50.1)	130 (3.1)	979 (23.5)	393 (9.4)	573 (13.8)	0 (0.0)	0.585	199 (4.8)	0.340
50–65	**4,613 (19.7)**	1,621 (35.1)	78 (1.7)	1,171 (25.4)	527 (11.4)	1,077 (23.3)	139 (3.0)		220 (4.8)	
65–74	**5,171 (22.1)**	1,258 (24.3)	63 (1.2)	1,098 (21.2)	506 (9.8)	1,929 (37.3)	317 (6.1)		277 (5.4)	
75–84	**5,539 (23.7)**	1,059 (19.1)	34 (0.6)	1,114 (20.1)	520 (9.4)	2,379 (42.9)	433 (7.8)		468 (8.4)	
≥85	**3,898 (16.7)**	657 (16.9)	38 (1.0)	756 (19.4)	404 (10.4)	1,728 (44.3)	315 (8.1)		400 (10.3)	
**Sex**										
Male	**10,979 (47.0)**	3,304 (30.1)	149 (1.4)	2,315 (21.1)	1044 (9.5)	3,553 (32.4)	614 (5.6)	0.080	796 (7.3)	0.085
Female	**12,404 (53.0)**	3,378 (27.2)	194 (1.6)	2,803 (22.6)	1306 (10.5)	4,133 (33.3)	590 (4.8)		768 (6.2)	
**Race or ethnicity**										
White, NH	**14,772 (63.2)**	3,817 (25.8)	162 (1.1)	3,236 (21.9)	1,367 (9.3)	5,304 (35.9)	886 (6.0)	0.362	1,076 (7.3)	0.199
Black, NH	**2,690 (11.5)**	1,157 (43.0)	73 (2.7)	598 (22.2)	266 (9.9)	525 (19.5)	71 (2.6)		117 (4.3)	
Hispanic	**2,708 (11.6)**	815 (30.1)	57 (2.1)	648 (23.9)	353 (13.0)	736 (27.2)	99 (3.7)		139 (5.1)	
Other,**^††^** NH	**2,115 (9.0)**	425 (20.1)	40 (1.9)	376 (17.8)	298 (14.1)	842 (39.8)	134 (6.3)		172 (8.1)	
Unknown	**1,098 (4.7)**	468 (42.6)	11 (1.0)	260 (23.7)	66 (6.0)	279 (25.4)	14 (1.3)		60 (5.5)	
**Chronic respiratory condition at discharge^§§^**										
No	**10,015 (42.8)**	3,085 (30.8)	147 (1.5)	2,179 (21.8)	980 (9.8)	3,142 (31.4)	482 (4.8)	0.092	604 (6.0)	0.092
Yes	**13,368 (57.2)**	3,597 (26.9)	196 (1.5)	2,939 (22.0)	1,370 (10.2)	4,544 (34.0)	722 (5.4)		960 (7.2)	
**Chronic nonrespiratory condition at discharge^¶¶^**										
No	**3,010 (12.9)**	1,243 (41.3)	53 (1.8)	690 (22.9)	226 (7.5)	748 (24.9)	50 (1.7)	0.242	174 (5.8)	0.058
Yes	**20,373 (87.1)**	5,439 (26.7)	290 (1.4)	4,428 (21.7)	2,124 (10.4)	6,938 (34.1)	1154 (5.7)		1,390 (6.8)	

**Abbreviations:** ICD-9 = *International Classification of Diseases, Ninth Revision*; ICD-10 = *International Classification of Diseases, Tenth Revision*; KPNC = Kaiser Permanente of Northern California; KPNW = Kaiser Permanente Northwest; N/A = not applicable; NH = non-Hispanic; PHIX = Paso del Norte Health Information Exchange; RT-PCR = reverse transcription–polymerase chain reaction; SMD = standardized mean or proportion difference.

* Hospitalizations with a discharge code consistent with COVID-19–like illness were included. COVID-19–like illness diagnoses included acute respiratory illness (e.g., respiratory failure or pneumonia) or related signs or symptoms (e.g., cough, fever, dyspnea, vomiting, or diarrhea) using diagnosis ICD-9 and ICD-10 codes. Clinician-ordered molecular assays (e.g., real-time RT-PCR) for SARS-CoV-2 occurring ≤14 days before to <72 hours after the encounter date were included.

**^†^** Partners contributing data on hospitalizations during dates of estimated ≥75% Omicron BA.1 predominance were in California (Dec 21, 2021–Mar 6, 2022), Colorado (Dec 25, 2021–Mar 12, 2022), Indiana (Dec 31, 2021–Mar 4, 2022), Minnesota and Wisconsin (Jan 1–Mar 5, 2022), New York (Dec 18, 2021–Feb 26, 2022), Oregon and Washington (Jan 1–Mar 12, 2022), Texas (Baylor Scott & White Health [Dec 18, 2021–Mar 5, 2022] and PHIX [Jan 8–Mar 19, 2022]), and Utah (Dec 27, 2021–Mar 19, 2022).

**^§^** Partners contributing data on hospitalizations during dates of estimated ≥75% Omicron BA.2/BA.2.12.1 predominance were in California (Mar 25–Jun 10, 2022), Colorado (Apr 9–Jun 4, 2022), Indiana (Mar 19–Jun 10, 2022), Minnesota and Wisconsin (Apr 9–Jun 4, 2022), New York (Mar 26–Jun 10, 2022), Oregon and Washington (Apr 9–Jun 10, 2022), Texas (Baylor Scott & White Health [Mar 26–Jun 4, 2022] and PHIX [Apr 23–Jun 10, 2022]), and Utah (Mar 28–Jun 10, 2022).

^¶^ Vaccination was defined as having received the listed number of doses of an mRNA-based COVID-19 vaccine within the specified range of number of days before the hospitalization index date, which was the date of respiratory specimen collection associated with the most recent positive or negative SARS-CoV-2 test result before the hospitalization or the admission date if testing only occurred after the admission.

** An absolute SMD ≥0.20 indicates a nonnegligible difference in variable distributions between hospitalizations for vaccinated versus unvaccinated patients or for patients with SARS-CoV-2–positive results versus those with SARS-CoV-2–negative results. For mRNA COVID-19 vaccination status, a single SMD was calculated by averaging the absolute SMDs obtained from pairwise comparisons of each vaccinated category versus unvaccinated; more specifically, as the average of the absolute value of the SMDs for 1) vaccinated with 2 doses 14–149 days earlier versus unvaccinated, 2) vaccinated with 2 doses ≥150 days earlier versus unvaccinated, 3) vaccinated with 3 doses 7–119 days earlier versus unvaccinated, 4) vaccinated with 3 doses ≥120 days earlier versus unvaccinated, and 5) vaccinated with 4 doses ≥7 days earlier versus unvaccinated.

**^††^** Other race includes Asian, Native Hawaiian or other Pacific islander, American Indian or Alaska Native, Other, and multiple races.

**^§§^** Chronic respiratory condition was defined as the presence of discharge code for asthma, chronic obstructive pulmonary disease, or other lung disease using ICD-9 and ICD-10 diagnosis codes.

^¶¶^ Chronic nonrespiratory condition was defined as the presence of discharge code for heart failure, ischemic heart disease, hypertension, other heart disease, stroke, other cerebrovascular disease, diabetes type I or II, other diabetes, metabolic disease, clinical obesity, clinically underweight, renal disease, liver disease, blood disorder, immunosuppression, organ transplant, cancer, dementia, neurologic disorder, musculoskeletal disorder, or Down syndrome using ICD-9 and ICD-10 diagnosis.

Among adults aged ≥50 years eligible to receive a fourth mRNA vaccine dose, VE for COVID-19–associated ED/UC encounters during the BA.2/BA.2.12.1 period was 32% at ≥120 days after the third dose (median interval = 170 days) but increased to 66% ≥7 days after the fourth dose (median interval = 28 days). VE against COVID-19–associated hospitalization was 55% ≥120 days after the third dose but increased to 80% ≥7 days after the fourth dose.

## Discussion

Data from the Omicron BA.1 sublineage surge in the United States during December 2021–February 2022 determined that VE was reduced compared with that during the previous Delta-predominant period (*6*). To date, estimates of differences in VE between the Omicron BA.1 and subsequent BA.2/BA.2.12.1 sublineage periods have been limited. In this estimate of VE against ED/UC visits and hospitalizations during the BA.1 and BA.2/BA.2.12.1 periods, VE declined during both periods ≥150 days after the second vaccine dose, highlighting the need for a third dose (i.e., the first booster) for eligible adults. Recent receipt of a third dose increased VE; however, some decline was observed ≥120 days after receipt of the dose. Among eligible adults aged ≥50 years, a fourth vaccine dose ≥120 days after receipt of the third dose improved VE during the BA.2/BA.2.12.1 period, although follow-up time after dose 4 was limited. These findings highlight the importance of staying up to date with COVID-19 vaccination, including recommended booster doses.

Although data on neutralizing antibodies have found BA.1 and BA.2 to be similar, recent data indicate slightly more immune escape for BA.2.12.1 (*1*). Data reported on Omicron sublineage VE have been limited. A study in the United Kingdom found inconsistent differences in VE for symptomatic COVID-19 and COVID-19–associated hospitalization, with higher VE against symptomatic COVID-19 but larger declines in VE against hospitalization observed during a period of BA.2 predominance versus a period of BA.1 predominance starting 10–14 weeks after a third COVID-19 vaccine dose (*7*). A study in Qatar found that after a second or third mRNA vaccine dose, declines in VE against symptomatic COVID-19 during BA.1 and BA.2 periods were similar, but the study did not identify enough severe cases to separate VE estimates by predominant variant (*8*). Differences between the current study and previous studies, including differences in proportions of persons with previous SARS-CoV-2 infection and the absence of BA.2.12.1 infections outside the United States might account for some variability in findings. After the BA.1 surge in the United States, a larger proportion of adults were found to have experienced a recent SARS-CoV-2 infection during the BA.2/BA.2.12.1 period, with antibody evidence of SARS-CoV-2 infection increasing from 33.5% in December 2021 to 57.7% by February 2022 (*9*). Unvaccinated persons were used as a referent group in VE analyses. If unvaccinated persons were more likely to have experienced recent infection, and infection-induced immunity provides some protection against re-infection, this could result in lower VE observed during the BA.2/BA.2.12.1 period. Although adults with documented past SARS-CoV-2 infection were excluded, infections are likely to be significantly underascertained because of lack of testing or increased at-home testing (*10*). In addition, although time since receipt of the second or third vaccine dose was stratified by time intervals, on average the time since vaccination was longer during the BA.2/BA.2.12.1 period. These differences were particularly pronounced in the analysis of ≥150 days after the second vaccine dose (median 289 days for hospitalized adults during the BA.1 period compared to 371 days during the BA.2/BA.2.12.1 period), which could account for some differences in VE estimates and highlights the importance of a third dose (first booster) for those who have not yet received it.

The findings in this analysis are subject to at least four limitations. First, previous SARS-CoV-2 infection was likely underascertained and might differentially affect observed VE during the BA.1 and BA.2/BA.2.12.1 periods. Second, residual confounding in VE estimates is possible. Third, no genetic characterization was available for SARS-CoV-2–positive specimens; therefore, date of testing was used to ascribe likely sublineage, and BA.2 and BA.2.12.1 sublineages were combined, given their overlap in circulation. Finally, this report did not assess VE against the most severe COVID-19–associated disease (e.g., respiratory failure) because of smaller numbers of these cases.

VE should continue to be monitored in the setting of newly emerging sublineages and variants, including Omicron sublineages BA.4 and BA.5, which became predominant in the United States in late June 2022. Eligible adults should stay up to date with recommended COVID-19 vaccinations, including a first booster dose for all eligible persons and second booster dose for adults aged ≥50 years. Booster doses should be obtained immediately when persons become eligible.

**Summary**

**What is already known about this topic?**

Little is known about COVID-19 vaccine effectiveness (VE) during the Omicron variant BA.2/BA.2.12.2–predominant period or VE of a fourth COVID-19 vaccine dose in persons aged ≥50 years.

**What is added by this report?**

VE during the BA.2/BA.2.12.2 period was lower than that during the BA.1 period. A third vaccine dose provided additional protection against moderate and severe COVID-19–associated illness in all age groups, and a fourth dose provided additional protection in eligible adults aged ≥50 years.

**What are the implications for public health practice?**

Immunocompetent persons should receive recommended COVID-19 booster doses to prevent moderate to severe COVID-19, including a first booster dose for all eligible persons and second dose for adults aged ≥50 years at least 4 months after an initial booster dose. Booster doses should be obtained immediately when persons become eligible.

## Footnotes

*
https://covid.cdc.gov/covid-data-tracker/#variant-proportions

† Funded by CDC, the VISION Network includes Baylor Scott & White Health (Texas), Columbia University Irving Medical Center (New York), HealthPartners (Minnesota and Wisconsin), Intermountain Healthcare (Utah), Kaiser Permanente Northern California (California), Kaiser Permanente Northwest (Oregon and Washington), Paso Del Norte Health Information Exchange-PHIX (Texas), Regenstrief Institute (Indiana), and University of Colorado (Colorado).

§ Medical events with a discharge code consistent with COVID-19–like illness were included. COVID-19–like illness diagnoses included acute respiratory illness (e.g., respiratory failure or pneumonia) or related signs or symptoms (e.g., cough, fever, dyspnea, vomiting, or diarrhea) using diagnosis codes from the *International Classification of Diseases, Ninth Revision* and *International Classification of Diseases, Tenth Revision*.

¶
https://www.cdc.gov/vaccines/covid-19/clinical-considerations/interim-considerations-us.html

** Local sequencing data were obtained in the states of participating VISION sites. Partners contributing data on medical events during dates of estimated ≥75% Omicron BA.1 predominance were in California (December 21, 2021–March 6, 2022), Colorado (December 25, 2021–March 12, 2022), Indiana (December 31, 2021–March 4, 2022), Minnesota and Wisconsin (January 1–March 5, 2022), New York (December 18, 2021–February 26, 2022), Oregon and Washington (January 1–March 12, 2022), Texas (Baylor Scott & White Health [December 18, 2021–March 5, 2022] and Paso del Norte Health Information Exchange [January 8–March 19, 2022]), and Utah (December 27, 2021–March 19, 2022).

†† Partners contributing data on medical events during dates of estimated ≥75% Omicron BA.2/BA.2.12.1 prevalence were in California (March 25–June 10, 2022), Colorado (April 9–June 4, 2022), Indiana (March 19–June 10, 2022), Minnesota and Wisconsin (April 9–June 4, 2022), New York (March 26–June 10, 2022), Oregon and Washington (April 9–June 10, 2022), Texas (Baylor Scott & White Health [March 26–June 4, 2022] and Paso del Norte Health Information Exchange [April 23–June 10, 2022]), and Utah (March 28–June 10, 2022).

§§ A booster (third) mRNA vaccine dose was first recommended by CDC for adults without immunocompromising conditions on September 23, 2021, and is currently recommended for all persons aged ≥5 years at least 5 months after a second mRNA vaccine dose. A second booster mRNA vaccine dose (fourth dose) was authorized for adults aged ≥50 years on March 29, 2022, at least 4 months after receiving a third mRNA vaccine dose. After this authorization CDC stated that adults aged ≥50 years may receive this additional booster dose; on May 19, 2022, CDC strengthened this recommendation to state that all adults aged ≥50 years should receive this additional booster dose. https://www.cdc.gov/vaccines/covid-19/clinical-considerations/interim-considerations-us.html

¶¶ Among ED/UC encounters and hospitalizations during the BA.1 period, 15,863 (11.3%) and 3,313 (11.8%), patients, respectively, had previous infection documented in their medical record and were excluded from analysis. Among ED/UC encounters and hospitalizations during the BA.2/BA.2.12.1 period, 17,293 (16.0%) and 3,829 (14.1%), patients, respectively, had previous infection documented in their medical record and were excluded from analysis.

*** The index date for each medical visit was defined as either the date of collection of a respiratory specimen associated with the most recent positive or negative SARS-CoV-2 test result before the medical visit or the date of the medical visit (if testing occurred only after the admission or visit date).

††† 45 C.F.R. part 46.102(l)(2), 21 C.F.R. part 56; 42 U.S.C. Sect. 241(d); 5 U.S.C. Sect. 552a; 44 U.S.C. Sect. 3501 et seq.
